# Individual and family factors correlated with children’s fruit consumption

**DOI:** 10.3389/fpubh.2024.1399704

**Published:** 2024-04-26

**Authors:** Xiangyi Wu, Yingjie Yu, Hairong He, Xiaohui Yu, Dandan Guo, Wenli Zhu

**Affiliations:** ^1^Department of Nutrition and Food Hygiene, School of Public Health, Peking University, Beijing, China; ^2^Beijing Center for Disease Prevention and Control, Beijing, China

**Keywords:** fruit consumption, children, food and nutrition literacy, family food environment, food frequency

## Abstract

**Background:**

Fruits are essential for health, yet their consumption in children is inadequate, with unclear influencing factors.

**Methods:**

A cross-sectional study was conducted among students in grades 3–12 in Beijing, China, from September 2020 to June 2021. Fruit consumption in children was surveyed using a self-administered food frequency questionnaire. Additionally, children’s food and nutrition literacy and family food environments were assessed using the “Food and Nutrition Literacy Questionnaire for Chinese School-age Children” and the “Family Food Environment Questionnaire for Chinese School-age Children,” respectively.

**Results:**

Out of 10,000 participating children, 62.5% consumed fruit daily, with a lower frequency among boys (59.3%) compared to girls (65.8%), and among senior students (48.6%) compared to junior (63.6%) and primary students (71.2%). Fruit consumption was positively associated with other healthy foods (vegetables, whole grains, etc.) and negatively with unhealthy foods (sugared soft drinks). Children with higher food and nutrition literacy consumed fruits daily more frequently (82.4% vs. 59.9%, *ORs* = 2.438, 95%*CI*: 2.072–2.868). A significant positive correlation was found between children’s fruit consumption and a healthy family food environment (66.4% vs. 50.2%, *OR* = 1.507, 95%*CI*: 1.363–1.667).

**Conclusion:**

The results indicate that individual food and nutrition literacy and family food environment are key positive predictors of children’s fruit consumption. Future interventions should focus on educating children and encouraging parents to foster supportive family environments.

## 1 Introduction

Fruits, as indispensable components of a healthy diet, offer numerous benefits throughout life, including gastrointestinal health protection, weight management, reduced risk of cardiovascular and metabolic diseases, defense against colorectal and lung cancers, enhanced psychological well-being, and increased bone mineral density in children and adults (1, 2). Global Burden of Disease Study 2019 suggested that dietary risk factors, especially high intake of sodium and low intake of whole grains and fruits were leading dietary risks for deaths and disability-adjusted life-years (DALYs) from non-communicable diseases (NCDs) worldwide (3). Globally, fruit consumption is far below the recommended levels (4–7). The Global School-Based Student Health Survey (GSHS) in 2019 reported that 10–30% of school-age students in approximately half of the countries did not consume any fruit per day (6). In China, less than 10% of children aged 2–19 consumed adequate fruit, with typical dietary intake being about one-third to one-sixth of recommended levels (7). Understanding the individual and environmental factors limiting fruit consumption, particularly in children, is crucial, as eating habits established in youth often persist into adulthood (8).

Actually, children’s food choices are complex outcomes influenced by individual characteristics (genetic, early-life, biological, demographic), cognition, emotion, and environmental factors ranging from the most proximal family influences to distal policy impacts (8–12). Food literacy, a key skill for understanding and making informed food decisions, is especially important at the individual level (13, 14). For instance, the Seoul Food Survey 2021 showed that the group with the highest quartile of food literacy scores was 1.83 times more likely to consume adequate servings of fruits compared to the lowest quartile group (15). However, Cochrane’s meta-analysis of 15 trials indicates the uncertainty of parent or child nutrition education interventions in effectively increasing fruit and vegetable consumption in children under five (16).

In terms of environmental factors, the family unit plays a crucial role in shaping children’s eating habits. Parents and caregivers are instrumental in planning and preparing family meals and act as role models, influencing their children’s eating behaviors through education and feeding practices (17–19). Systematic reviews have highlighted strong correlations between food availability, parental modeling, and children’s fruit consumption (18, 20). Feeding patterns adopted by caregivers, especially those that are encouraging and well-regulated, have been associated with higher fruit intake in children. Conversely, instrumental and/or emotional feeding patterns often correlate with inadequate fruit consumption (21–23). Grandparents typically tend to be more permissive or indulgent, whereas parents are more likely to use gentle persuasion or rewards (21). However, some studies have challenged these findings, suggesting that family interventions have a lesser impact on children’s fruit consumption (12). Additionally, the Cochrane meta-analysis indicates that there is insufficient evidence to firmly conclude that caregiver involvement significantly improves children’s dietary habits (22).

Overall, fruit consumption is influenced by multiple factors that interact with each other, meaning they cannot be considered in isolation. Previous studies focusing on determining factors of children’s fruit consumption often examined individual elements, potentially leading to an incomplete understanding of the true impact of each factor. Additionally, only a few studies have specifically focused on the Chinese population. Cultural differences in parent–child relationships may affect these findings, thus limiting their applicability beyond the studied demographic. Furthermore, there is a lack of consistency in measuring both individual and environmental factors. As fruit consumption patterns can differ from those of vegetables, it is important to study them independently. This study aims to explore the effects of both individual and family environmental factors on fruit consumption among primary and secondary students in Beijing, China, to provide scientific evidences to promote fruit consumption.

## 2 Materials and methods

### 2.1 Study design and sampling

A cross-sectional study was carried out in Beijing, China, from September 2020 to June 2021. The targeted group consisted of primary and secondary students in Beijing, covering grades 3–12, with a general range of 8–18 years old. Specifically, this included primary school students in grades 3–6 (aged 8–12), junior high school students in grades 6–9 (aged 12–15), and senior high school students in grades 9–12 (aged 15–18). Participants were selected using a multi-stage stratified cluster sampling strategy, as detailed in the literature (24). Based on the study design and sampling method, the sample size calculation formula for each stratum was as follows:

$$n=\frac{\mu^{2}\pi \left(1-\pi \right)}{\delta^{2}}$$

Due to the lack of representative level on food and nutrition literacy, the sample size for this survey was estimated based on health literacy level. According to the Beijing Municipal Health Commission, the health literacy level of residents in Beijing was 32.3% in 2018 (25). Taking μ = 1.96, δ = 0.15π, the calculated sample size was n = 358. The survey participants were stratified into three strata by school level (primary school, junior high school, senior high school), two strata by urbanization (central urban area, rural area), and two strata by gender (male, female), making a total of 12 strata. Assuming an estimated 10% for invalid questionnaires and non-response, with a design effect (DEFF) of 2 for the multistage cluster sampling, the total sample size was calculated to be 9,547 individuals.

The study’s protocol was thoroughly explained to potential participants and their caregivers during parent-teacher meetings. Subsequently, informed written consent was voluntarily obtained from 10,000 child-caregiver pairs, resulting in a response rate of 98.8%.

The study received approval from the Institutional Review Board of the Beijing Center for Disease Prevention and Control (approval number 2020-29) and was conducted in compliance with the Declaration of Helsinki. Measures were taken to ensure the privacy of the child-caregiver pairs participating in the study and to maintain the confidentiality of their personal information.

### 2.2 Investigation of child’s fruit and other foods consumption

Food consumption was assessed using a self-administered short food frequency questionnaire (26, 27), which included 10 items: (1) Fruits (excluding fruit juice); (2) Vegetables; (3) Whole grains; (4) Dairy products (excluding milk beverages); (5) Legume products; (6) Fish (aquatic animals); (7) Breakfast; (8) Sugared soft drinks; (9) Fried food; (10) Fast food. Participants reported their food consumption frequency over the previous 7 days using categorical responses ranging from ‘None’, ‘1–2 days’, ‘3–4 days’, ‘5–6 days’, to ‘Daily’. Additionally, the diversity of foods consumed in the past 24 h was recorded to assess dietary diversity. The questionnaire completion was facilitated by investigators in the classroom setting.

According to the Dietary Guidelines for Chinese Residents (2022), it is recommended to consume fruits, vegetables, whole grains, dairy products, legumes, and have breakfast ‘daily’; fish should be eaten ‘weekly (at least once a week)’, while the consumption of sugared soft drinks, fried food, and fast food should be ‘none’ (28). Dietary diversity was defined as consuming more than 12 different types of food.

### 2.3 Food and nutrition literacy assessment of children

Children’s food and nutrition literacy was assessed using “Food and Nutrition Literacy Questionnaire for Chinese School-age Children (FNLQ-SC),” which was developed and validated previously by our research group (13).

FNLQ-SC comprises four dimensions including: food and nutrition related knowledge; and skills in selecting, preparing food, and healthy eating. The FNLQ-SC demonstrated acceptable internal consistency (Cronbach’s α = 0.698) and an acceptable fit in general (RMSEA = 0.70) (13), which has been revised and used in Turkish school age adolescents (29).

Students self-administered the FNLQ-SC questionnaire in the classroom, guided by investigators.

### 2.4 Family food environment assessment and demographic characteristics measurement

Family food environment was assessed by the “Family Food Environment Questionnaire for Chinese School-age Children (FFEQ-SC),” previously developed and validated by our group (24, 30).

The conceptual framework of FFEQ-SC was primarily based on the Analysis Grid for Elements Linked to Obesity (ANGELO) (31). This framework encompasses various environmental dimensions including physical, economic, policy, and sociocultural aspects. The FFEQ-SC is composed of six key dimensions: (1) Family Socioeconomic Status (SES), which includes factors like family food expenditure and overall family affluence status (32); (2) Food Availability (FA), covering the availability of both healthy foods (including fruits) and unhealthy foods (such as sugar-sweetened beverages); (3) Feeding Patterns (FP), encompassing approaches like permission, restriction, enforcement, role modeling, and encouragement; (4) Health-oriented Family Food Rules (FR); (5) Family Meal Practices (MP); and (6) Caregiver’s Food Literacy (CFL). Further details on these dimensions are provided in the literature (24).

Caregivers completed the FFEQ-SC questionnaire at home, with investigators available for questions via telephone. Additional demographic data were collected, including students’ grade, gender, caregivers’ education level, household income, and family size.

### 2.5 Anthropometric data

Anthropometric data of children, including height and weight, were obtained from the “Beijing School Health Information Management System” with permissions from participating schools and individuals. Body mass index (BMI) was calculated as weight in kilograms divided by the square of the height in meters (kg/m^2^), and children’s weight status was assessed based on Chinese standards for school-age children and adolescents (33, 34). Children with BMI below the cutoff corresponding to their gender and age were categorized as wasted, and over as overweight (included obese).

### 2.6 Variables value assignment and statistical analysis

Data were processed and analyzed using EpiData 3.1 and SPSS version 27.0.

The Food and Nutrition Literacy Questionnaire for Chinese School-age Children (FNLQ-SC) consists of 35 questions, totaling a maximum score of 72 points. This includes dimensions on food and nutrition-related knowledge (20 points), skills in food selection (20 points), food preparation (10 points), and healthy eating (22 points). For grades 3–4, the questionnaire is slightly modified, comprising 31 questions with a total of 64 points, while for grades 5–6, it includes 34 questions totaling 70 points. To facilitate comparisons across different age groups, the total scores for all grades are converted to a centesimal (percentage) system. There was no recognized cut-off score, the study defines ‘nutritionally literate’ as a score exceeding the 80th percentile of the total score, which corresponds to 22.2 for knowledge, 9.6 for food selection, 10.8 for food preparation, 14.4 for eating, and an overall score of 80.

The Family Food Environment Questionnaire for Chinese School-age Children (FFEQ-SC) comprises 49 questions, with a scoring range from 0 to 100. The questionnaire evaluates several dimensions, including Socioeconomic Status (SES), Food Availability (FA), Feeding Patterns (FP), Food Rules (FR), Meal Practices (MP), and Caregiver’s Food Literacy (CFL). The scoring ranges for these dimensions are 0 to 8 for SES, 0 to 16 for FA, 0 to 18 for FP, 0 to 24 for FR, 0 to 17 for MP, and 0 to 17 for CNL, respectively, with higher scores indicating healthier environments. There was no recognized cut-off score, a ‘healthy family food environment’ was defined as scoring above the 60th percentile of the total score, equating to scores of 4.8 for SES, 9.6 for FA, 10.8 for FP, 14.4 for FR, 10.2 for MP, and 10.2 for CNL, with an overall questionnaire score of 60 or more.

Descriptive statistics were used to depict the distribution of fruit consumption. Differences in the frequency of fruit consumption were compared across demographic characteristics, food and nutrition literacy, and categories of family food environment using the Chi-square test. Binary logistic regression models were employed to explore the relationships between the frequency of food consumption and factors specific to the child and their family. Odds ratios (*ORs*) with 95% confidence intervals (*CIs*) were computed for the independent variables. Approximately 96.8% (*n* = 9,678) of the study sample had complete data for confounding variables and entered the multivariate analysis. Missing values were not imputed. The sample excluding participants with missing data (*n* = 9,678) was found to have similar daily fruit consumption compared with the study sample (*n* = 1,000). The threshold for statistical significance was set at a *p*-value of 0.05.

## 3 Results

### 3.1 Demographic characteristics of children’s fruit consumption

Among 10,000 students aged 7–19, the distribution across primary (grade 3–6), junior (grade 7–9), and senior (grade 10–12) high school was 41.8, 29.9, and 28.3%, respectively, with 49.6% female. Most caregivers (97.3%) were parents, and a third had college-level education or higher. Details in Table 1.

**Table 1:** Demographic characteristics of child’s fruit consumption.

Group	*N* (%)	Fruit consumption during the past 7 days *n* (%)					*ORs*(95%*CI*) of daily consumption
		None	1–2 days	3–4 days	5–6 days	Daily	
**Total**	10,000(100.0)	204(2.0)	880(8.8)	1,424(14.2)	1,241(12.4)	6,251(62.5)	
**Child**							
Grade							
3 ~ 6	4,176(41.8)	41(1.0)	225(5.4)	432(10.3)	506(12.1)	2,972(71.2)	–
7 ~ 9	2,991(29.9)	42(1.4)	242(8.1)	436(14.6)	370(12.4)	1901(63.6)	0.707(0.639–0.781)*
10 ~ 12	2,833(28.3)	121(4.3)	413(14.6)	556(19.6)	365(12.9)	1,378(48.6)	0.384(0.347–0.424)*
Gender							
Male	5,042(50.4)	140(2.8)	481(9.5)	776(15.4)	655(13.0)	2,990(59.3)	–
Female	4,958(49.6)	64(1.3)	399(8.0)	648(13.1)	586(11.8)	3,261(65.8)	1.319(1.216–1.430)*
BMI							
Normal	5,278(52.8)	113(2.1)	460(8.7)	750(14.2)	645(12.2)	3,310(62.7)	–
Wasted	517(5.2)	14(2.7)	44(8.5)	71(13.7)	76(14.7)	312(60.3)	0.905(0.752–1.089)
Overweight	4,082(40.8)	72(1.8)	367(9.0)	588(14.4)	500(12.2)	2,555(62.6)	0.995(0.914–1.082)
District							
Urban	3,542(35.4)	45(1.3)	233(6.6)	469(13.2)	485(13.7)	2,310(65.2)	–
Rural	6,458(64.6)	159(2.5)	647(10.0)	955(14.8)	756(11.7)	3,941(61.0)	0.835(0.767–0.909)*
**Household**							
Number of children							
1	6,281(62.8)	120(1.9)	548(8.7)	886(14.1)	774(12.3)	3,953(62.9)	–
≥2	3,638(36.4)	78(2.1)	325(8.9)	522(14.3)	462(12.7)	2,251(61.9)	0.956(0.879–1.040)
Principal caregiver							
Parents	9,727(97.3)	192(2.0)	855(8.8)	1,385(14.2)	1,212(12.5)	6,093(62.5)	–
Grandparents	166 (1.7)	4(2.4)	15(9.0)	18(10.8)	19(11.4)	110(66.3)	1.177(0.851–1.628)
Caregiver’s educational level							
≤Junior high school	1,625(16.5)	75(4.5)	221(13.4)	328(19.9)	214(13.0)	814(49.3)	–
High school	2,545(25.5)	59(2.3)	280(11.0)	406(16.0)	310(12.2)	1,490(58.5)	1.454(1.284–1.647)*
Junior college	2,437(24.4)	35(1.4)	189(7.8)	322(13.2)	289(11.9)	1,602(65.7)	1.975(1.738–2.244)*
≥College	3,285(32.9)	29(0.9)	183(5.6)	352(10.7)	423(12.9)	2,298(70.0)	2.397(2.122–2.708)*
Household income *per capita* annually (*yuan*)							
<20,000	1731(17.3)	48(2.8)	215(12.4)	308(17.8)	229(13.2)	931(53.8)	–
20,000 ~ 39,999	2,181(21.8)	61(2.8)	215(9.9)	335(15.4)	279(12.8)	1,291(59.2)	1.246(1.097–1.416)*
40,000 ~ 69,999	2,599(26.0)	42(1.6)	231(8.9)	366(14.1)	318(12.2)	1,642(63.2)	1.474(1.303–1.668)*
≥70,000	3,398(34.0)	47(1.4)	211(6.2)	397(11.7)	409(12.0)	2,334(68.7)	1.885(1.673–2.123)*

*p < 0.05; Total percentage for some variables was less than 100% because of missing data. Chi-square test was employed.

62.5% of children consumed fruit on a daily basis, while more than one third failed to meet the recommendation. Children’s daily fruit consumption was negatively correlated with grade level, while positively correlated with caregiver’s education level and household income. Notably, the frequencies of daily fruit consumption in junior and senior high school students were significantly lower than in primary school students (48.6, 63.6, and 71.2% respectively, *p* < 0.05). Girls consumed fruits on a daily basis more frequently than boys (65.8% vs. 59.3%, *OR* = 1.319, 95%*CI*: 1.216–1.430), and urban children had a higher fruit consumption frequency (65.2%) compared to rural children (61.0%, *OR* = 0.835, 95%*CI*: 0.767–0.909). Additionally, it was observed that children consumed fruit more frequently with increasing caregiver’s education level and higher household income (*p* < 0.05).

### 3.2 Relation of children’s fruit consumption with food and nutrition literacy

11.7% of participant students were considered nutritionally literate, showing a strong correlation with daily fruit consumption. As shown in Table 2.

**Table 2:** Child’s fruit consumption according to individual food and nutrition literacy.

Dimensions of food and nutrition literacy	*N* (%)	Fruit consumption during the past 7 days, *n* (%)					*ORs*(95%*CI*) of daily consumption
		None	1–2 days	3–4 days	5–6 days	Daily	
Total score							
Mean ± SD	68.4 ± 9.8	60.4 ± 10.7^a^	63.1 ± 9.0^b^	65.1 ± 9.2^c^	67.3 ± 9.4^d^	70.4 ± 9.5^e^	
<80	8,829(88.3)	203(2.3)	862(9.8)	1,344(15.2)	1,134(12.8)	5,286(59.9)	–
≥80	1,171(11.7)	1(0.1)	18(1.5)	80(6.8)	107(9.1)	965(82.4)	2.610(2.226–3.060)*
Knowledge							
Mean ± SD	19.9 ± 3.6	18.2 ± 4.4^a^	18.7 ± 3.5^ab^	19.1 ± 3.5^b^	19.7 ± 3.5^c^	20.3 ± 3.6^d^	
<22.2	7,070(70.7)	163(2.3)	731(10.3)	1,130(16.0)	905(12.8)	4,141(58.6)	–
≥22.2	2,930(29.3)	41(1.3)	149(5.1)	294(10.0)	336(11.5)	2,110(72.0)	1.993(1.809–2.193)*
Food selection							
Mean ± SD	18.5 ± 3.5	16.2 ± 3.7^a^	17.1 ± 3.4^b^	17.6 ± 3.5^c^	18.2 ± 3.4^d^	19.1 ± 3.4^e^	
<22.2	8,484(84.8)	190(2.2)	823(9.7)	1,278(15.1)	1,088(12.8)	5,105(60.2)	–
≥22.2	1,516(15.2)	14(0.9)	57(3.8)	146(9.6)	153(10.1)	1,146(75.6)	1.871(1.648–2.125)*
Food preparation							
Mean ± SD	9.7 ± 2.1	8.5 ± 2.5^a^	8.9 ± 2.0^a^	9.2 ± 1.9^b^	9.5 ± 2.0^c^	10.0 ± 2.1^d^	
<11.1	7,367(73.7)	169(2.3)	754(10.2)	1,175(15.9)	978(13.3)	4,291(58.2)	–
≥11.1	2,633(26.3)	35(1.3)	126(4.8)	249(9.5)	263(10.0)	1960(74.4)	1.867(1.687–2.066)*
Eating							
Mean ± SD	20.3 ± 4.3	17.6 ± 4.9^a^	18.4 ± 4.1^a^	19.2 ± 4.1^b^	19.9 ± 4.2^c^	21.1 ± 4.2^d^	
<24.5	8,338(83.4)	191(2.3)	825(9.9)	1,305(15.7)	1,078(12.9)	4,939(59.2)	–
≥24.5	1,662(16.6)	13(0.8)	55(3.3)	119(7.2)	163(9.8)	1,312(78.9)	2.140(1.882–2.435)*

*p < 0.05, adjusted for students’ grade and gender; a, b, c, d, e indicated significant differences between groups (*p* < 0.05). Chi-square test was employed.

The children with higher food and nutrition literacy level, including more food and nutrition related knowledge and skills, consumed fruit daily more frequently (82.4% vs. 59.9%, *OR* = 2.610, 95%*CI*: 2.226–3.060). Moreover, as the frequency of fruit consumption increased, the score of food and nutrition literacy increased significantly (*p* < 0.05), the children consumed fruit daily had a score of 70.4(±9.5) compared with those did not eat fruit during the past 7 days (60.4 ± 10.7, *p* < 0.05).

### 3.3 Interrelation of children’s fruit consumption with other foods consumption behaviors

The results showed that children’s dietary intake of healthy foods, including fruits, was below recommendations. The percentages of daily consumption of vegetable, whole grain, dairy, legumes, and breakfast were 71.5, 27.1, 54.9, 13.2 and 78.8% respectively, while more than two thirds of children consumed soft drinks and fried foods weekly, and about half of children eat fast food at least once a week. And only 21.1% reached up to the dietary diversity. As shown in Table 3.

**Table 3:** Interrelation of child’s fruit consumption with other foods consumption behaviors.

Food consumption during the past 7 days	*N* (%)	Fruit consumption during the past 7 days, *n* (%)		*ORs*(95%*CI*) of daily consumption
		≤6 days	Daily	
Vegetables				
≤6 days	2,826(28.3)	1774(62.8)	1,052(37.2)	–
Daily	7,147(71.5)	1961(27.4)	1,052(72.6)	4.629(4.209–5.091)*
Whole grains				
≤6 days	7,291(72.9)	3,107(42.6)	4,184(57.4)	–
Daily	2,705(27.1)	641(23.7)	2064(76.3)	2.435(2.199–2.697)*
Dairy products				
≤6 days	4,508(45.1)	2,359(52.3)	2,149(47.7)	–
Daily	5,488(54.9)	1,388(25.3)	4,100(74.7)	3.354(3.075–3.657)*
Legumes				
≤6 days	8,676(86.8)	3,547(40.9)	5,129(59.1)	–
Daily	1,322(13.2)	201(15.2)	1,121(84.8)	4.338(3.698–5.089)*
Fish				
None	2,734(27.3)	1,315(48.1)	1,419(51.9)	–
Weekly	7,263(72.6)	2,432(33.5)	4,831(66.5)	1.911(1.743–2.094)*
Breakfast				
≤6 days	2,110(21.1)	1,236(58.6)	874(41.4)	–
Daily	7,882(78.8)	2,511(31.9)	5,371(68.1)	2.675(2.418–2.959)*
Sugared soft drinks				
None	2,911(29.1)	918(31.5)	1993(68.5)	–
Weekly	7,080(70.8)	2,829(40.0)	4,251(60.0)	0.786(0.716–0.863)*
Fried food				
None	3,265(32.7)	1,102(33.8)	2,163(66.2)	–
Weekly	6,730(67.3)	2,646(39.9)	4,084(60.7)	0.889(0.813–0.973)*
Fast food				
None	4,716(47.2)	1736(36.8)	2,980(63.2)	–
Weekly	5,281(52.8)	2013(38.1)	3,268(61.9)	1.001(0.921–1.087)
Dietary diversity				
<12	7,866(78.8)	3,168(40.3)	4,698(59.7)	–
≥12	2,112(21.1)	574(27.7)	1,538(72.8)	1.833(1.646–2.042)*

*p < 0.05, adjusted for students’ grade and gender; “weekly” means food consumption at least once a week. Binary logistic regression models were employed.

Eating behaviors were interrelated, with positive correlations between fruit consumption and other healthy foods (vegetable, whole grain, dairy, legumes, and fish), and negative correlations with unhealthy foods (sugared soft drinks and fried foods). Children consuming vegetable daily also consumed fruits more frequently (72.6% vs. 37.2%, *OR* = 4.629, 95%*CI*: 4.209–5.091). And the children drinking sugared soft drinks weekly consumed fruits much lower (60.0% vs. 68.5%, *OR* = 0.786, 95%*CI*: 0.716–0.863).

### 3.4 Relation of children’s fruit consumption with family food environment

In the study, 74.2% of the participating students were from families with a healthier food environment. Notably, 76.8% of these families ensured the availability of healthy foods at home, 65.2% had health-oriented food rules, 84.3% practiced good meal habits, and 78.7% of caregivers were nutritionally literate. However, it is important to highlight that only 12.0% of families adopted an encouragement style in feeding their children.

A healthier family food environment was significantly linked to increased fruit consumption among the children. For students from families with a healthier food environment (scoring ≥60 points), the likelihood of daily fruit consumption was 70% higher (*OR* = 1.704, 95%*CI*: 1.544–1.879) compared to those from less healthy environments. Children from families with a higher socioeconomic status, better access to healthy foods, encouragement-based feeding patterns, and healthier meal practices were more likely to consume fruit (*p* < 0.05). Further details are available in Table 4.

**Table 4:** Child’s fruit consumption according to family food environment.

Dimensions of family food environment	*N* (%)	Fruit consumption during the past 7 days *n* (%)					*ORs*(95%*CI*) of daily consumption
		None	1–2 days	3–4 days	5–6 days	Daily	
Total score							
Mean ± SD	65.7 ± 8.4	61.2 ± 8.6^a^	62.2 ± 8.5^a^	63.7 ± 8.3^b^	65.3 ± 8.6^c^	66.8 ± 8.2^d^	
<60	2,267(22.7)	82(3.6)	317(14.0)	425(18.7)	305(13.5)	1,138(50.2)	–
≥60	7,419(74.2)	113(1.5)	530(7.15)	948(12.8)	903(12.2)	4,925(66.4)	1.704(1.544–1.879)*
Family socioeconomic status							
Mean ± SD	4.3 ± 1.3	4.2 ± 1.2^ab^	4.1 ± 1.3^a^	4.2 ± 1.3^a^	4.2 ± 1.4^a^	4.3 ± 1.3^b^	
<4.8	6,113(61.1)	135(2.2)	575(9.4)	924(15.1)	763(12.5)	3,716(60.8)	–
≥4.8	3,785(37.9)	63(1.7)	295(7.8)	480(12.7)	472(12.5)	2,475(65.4)	1.194(1.095–1.302)*
Family food availability							
Mean ± SD	11.4 ± 2.2	10.5 ± 2.1^a^	10.6 ± 2.2^a^	10.9 ± 2.1^b^	11.3 ± 2.1^c^	11.7 ± 2.1^d^	
<9.6	2,217(22.2)	74(3.3)	310(14.0)	416(18.8)	297(13.4)	1,120(50.5)	–
≥9.6	7,677(76.8)	122(1.6)	561(7.3)	987(12.9)	939(12.2)	5,068(66.0)	1.670(1.514–1.842)*
Family feeding patterns							
Mean ± SD	8.7 ± 1.9	8.2 ± 2.2^a^	8.5 ± 1.9^ab^	8.5 ± 1.9^b^	8.7 ± 1.8^c^	8.8 ± 1.9^c^	
<10.8	8,699(87.0)	178(2.0)	771(8.9)	1,271(14.6)	1,096(12.6)	5,383(61.9)	–
≥10.8	1,196(12.0)	20(1.7)	99(8.3)	133(11.1)	137(11.5)	807(67.5)	1.187(1.042–1.354)*
Family food rules							
Mean ± SD	16.2 ± 4.5	14.9 ± 5.3^ab^	15.0 ± 5.0^a^	15.7 ± 4.5^b^	16.0 ± 4.6^b^	16.5 ± 4.4^c^	
<14.4	3,374(33.7)	91(2.7)	399(11.8)	549(16.7)	434(12.9)	1901(56.3)	–
≥14.4	6,533(65.2)	107(1.6)	472(7.2)	855(13.1)	798(12.2)	4,290(65.8)	1.286(1.177–1.404)*
Family meal practices							
Mean ± SD	12.3 ± 1.9	11.7 ± 2.2^a^	11.7 ± 1.9^a^	12.0 ± 1.9^b^	12.2 ± 1.9^c^	12.5 ± 1.9^d^	
<10.2	1,445(14.5)	47(3.3)	205(14.2)	250(17.3)	183(12.7)	760(52.6)	–
≥10.2	8,426(84.3)	151(1.8)	661(7.8)	1,150(13.6)	1,047(12.4)	5,417(64.3)	1.415(1.260–1.588)*
Caregiver’s food and nutrition literacy							
Mean ± SD	12.8 ± 2.9	11.7 ± 2.9^a^	12.1 ± 3.0^b^	12.4 ± 2.9^c^	12.7 ± 2.9^d^	13.0 ± 2.8^e^	
<10.2	1944(19.4)	61(3.1)	236(12.1)	313(16.1)	243(12.5)	1,091(56.1)	–
≥10.2	7,871(78.7)	136(1.7)	627(8.0)	1,079(13.7)	978(12.4)	5,051(64.2)	1.409(1.271–1.562)*

*p < 0.05, adjusted for students’ grade and gender; a, b, c, d, e indicated significant differences between groups (*p* < 0.05). Chi-square test was employed.

### 3.5 Multiple regression analysis predicting children’s fruit consumption

Regression analysis (Table 5) revealed that both individual food and nutrition literacy and family food environment were significant predictors of children’s fruit consumption, even after adjusting for demographic characteristics. The children with higher food and nutrition literacy were more likely to consume fruit (*B* = 0.891, *OR* = 2.438, 95%*CI*: 2.072–2.868), so were those with healthier family food environment (*B* = 0.410, *OR* = 1.507, 95%*CI*: 1.363–1.667).

**Table 5:** Logistic regression analysis of child’s daily fruit consumption.

Independent variable	*B*	*P*	*ORs* (95%*CI*)
(Constant)	−0.514	0.015*	
Grade			
3 ~ 6	–		–
7 ~ 9	−0.148	<0.006*	0.863(0.776–0.959)
10 ~ 12	−0.736	<0.001*	0.479(0.430–0.533)
Gender (Female)	0.290	<0.001*	1.336(1.226–0.457)
**Food and nutrition literacy (≥80)**	**0.891**	<0.001*	**2.438(2.072–2.868)**
District (Rural)	0.013	0.795	1.013(0.920–1.115)
Number of children (≥2)	−0.071	0.130	0.931(0.849–1.021)
Principal caregiver (grandparents)	0.000	1.000	1.000(0.772–1.296)
Caregiver’s education			
≤Junior high school	—		–
High school	0.282	<0.001*	1.325(1.159–1.515)
Junior college	0.468	<0.001*	1.597(1.387–1.839)
≥College	0.559	<0.001*	1.749(1.514–2.021)
Household income *per capita* (yuan)			
<20,000	–		–
20,000 ~ 39,999	0.118	0.087	1.125(0.983–1.287)
40,000 ~ 69,999	0.183	0.007*	1.201(1.051–1.372)
≥70,000	0.300	<0.001*	1.349(1.178–1.545)
**Family food environments (≥60)**	**0.410**	<0.001*	**1.507(1.363–1.667)**

*p < 0.05. Binary logistic regression models were employed.

## 4 Discussion

A cross-sectional investigation of students in grade 3–12 in Beijing, China, revealed less than two thirds of them consumed fruit daily. This frequency of fruit consumption was found to be associated with various individual and family factors. Children with higher levels of food and nutrition literacy and a healthier family food environment were more likely to consume fruit regularly. It was worth noting that boys, who were in higher grades, had caregivers with lower levels of education and lower household incomes, should be targeted for interventions to promote their fruit intake.

Eating fruit offers significant health benefits for children and adolescents, yet global intake levels are generally low (35, 36). The China Nutrition and Health Survey (CNHS) from 2010 to 2012 showed that only 40.8% of children aged 6–17 consumed fruit daily, and 67.5% did not meet the recommended intake of 150 g (37). Fruit consumption was notably higher in urban than rural areas, with 57.2–59.0% of children in metropolitan cities eating fruit daily (37), aligning with this study’s finding of 62.5% daily consumption among children in Beijing. The Health Behaviour in School-aged Children survey (HBSC), conducted every 4 years since 1986 under the WHO Regional Office for Europe, also informed this study’s fruit consumption assessment method (26). Considering the recall errors of children and adolescents, the Food Frequency Questionnaire (FFQ) was administrated for the food intakes during the past week, despite the potential bias associated with this time frame. The HBSC data indicated a general increase in daily fruit consumption among adolescents from 2002 to 2018 in Western European countries. Overall, daily consumption remained low at 36.4%, but was higher among girls (39.9% vs. 32.7% among boys), younger adolescents (42.1% in 11-year-olds vs. 35.4% in 13-year-olds and 31.3% in 15-year-olds), and in the high Family Affluence Scale (FAS) group (42.6% vs. 36.1% in the medium and 31.7% in the low FAS groups) (35). This study observed a similar trend in children’s fruit consumption, with lower rates among boys, older children, and those from lower socio-economic backgrounds. The UK National Diet and Nutrition Survey suggested a decline in fruit intake starting from the age of 7 years, reaching the lowest level during adolescence; by 17 years, boys consumed approximately 0.93 fewer fruit portions than at the age of 2 years (38). In this study, senior high school students had the lowest frequency of fruit consumption compared to primary students (48.6% vs. 71.2%, *OR* = 0.384). As children age, the decreased family influence on eating behaviors and the increased autonomy in food selection may account for the decrease in fruit consumption (39). However, interestingly, vegetable consumption remains relatively stable throughout childhood and adolescence, despite the decline in fruit intake (37). This stark contrast between fruit and vegetable consumption may stem from the fact that vegetables are often served as part of the main meal, making them less prone to being skipped or avoided compared to fruit. Current evidence does not strongly indicate a resurgence in fruit consumption during early adulthood. Otherwise, the girls and lower graders often had higher food and nutrition literacy (13), which was positively correlated with fruit consumption. Therefore, initiatives aimed at promoting increased fruit intake should carefully consider these observed trends related to age, gender, and socio-economic disparities.

Improving dietary habits requires individuals to possess food-related skills and abilities and a comprehension of the social context. A systematic review showed that food literacy may play a crucial role in shaping adolescent’s dietary intake (14). This study employed a recognized and validated assessment instrument of food and nutrition literacy to explore its correlation with children’s fruit consumption. The results revealed that 11.7% of the children were nutritionally literate, which was strongly correlated with daily fruit consumption (*OR* = 2.438, 95%*CI*: 2.072–2.868); as the frequency of fruit consumption increased, the food and nutrition literacy score increased significantly (*p* < 0.05). Other studies arrived at similar conclusions, demonstrating that better cooking and food skills were associated with healthier eating behaviors and greater fruit consumption for boys and girls (15, 40). These findings underscore the importance of enhancing food and nutrition literacy among children, and public health interventions should focus on improving food and nutrition-related knowledge and skills to enhance child’s fruit consumption. Nevertheless, studies have shown that nutrition education can improve food and nutrition literacy but has no significant impact on fruit and other foods consumption (16, 41). In reality, food consumption is influenced by individual and environmental factors, which can be better understood through a socio-ecological model (42).

The family is the most immediate environment influencing a child’s food intake, encompassing home food accessibility, family meals, role modeling, and feeding patterns. This study employed a validated instrument to systematically assess the family food environment’s impact on children’s fruit consumption. The results indicated a significant positive association between children’s daily fruit consumption and their family food environment (*OR* = 1.507, 95%*CI*: 1.363–1.667). Children from families with easy access to healthy foods, encouragement-based feeding patterns, healthy food rules, positive meal practices, and caregivers with higher food literacy were more likely to consume fruit (*p* < 0.05). Similar findings have been observed in other studies, where adolescents from families not receiving supplemental food assistance, and those whose parents/caregivers reported higher fruit consumption, availability, encouragement, and set rules for fruit consumption, tended to eat more fruits (17–22, 43). Parental modeling and active guidance have been identified as strong predictors of healthy food consumption. In contrast, pressure to eat and restrictive feeding practices may undermine children’s autonomous motivation and self-regulation in eating (23, 43–45). In China, parents often pressure children to eat more to ensure adequate nutrient intake, driven by their psychological needs, leading to anxiety if children consume less than expected (46). Behavioral theory suggests that criticism or punishment during meals can instill a negative attitude towards eating in children, potentially leading to eating disorders. However, another study offers a contrasting view, indicating that children with authoritative and permissive fathers, as well as girls with authoritative mothers at ages 4–5, are more likely to consume fruits in later years (47).

One limitation of this study is the chosen methodology for assessing food consumption, particularly in relation to its suitability for younger participants and its potential time frame bias. As younger children often struggle to accurately recall their dietary intake, alternative assessment methods [such as including parents as proxy respondents (48)] should be considered, particularly for younger participants, to ensure the accuracy and validity of dietary data in research studies. Existing studies often have limitations, primarily due to their cross-sectional design, which restricts the ability to generalize the results. Additionally, the methods of assessing environmental factors and food consumption pose further challenges. While this study comprehensively examined individual and environmental factors influencing fruit consumption, it did not take into account aspects of the food system, such as sensory and physical properties, variety, visibility, taste, satiety value, preparation methods, and time costs. Moreover, other socio-ecological environmental factors, including schools, peers, neighborhoods, and broader macro-environmental influences, were not considered. Therefore, based on follow-up cohort design, a complex study, including individual (physiological and psychological factors, knowledge, belief and skill, and early-life food exposure) and socio-ecological food environmental factors (physical and social factors of family, peers, school, neighborhoods, food marketing, policy and international trade) would be undertaken to explore their comprehensive effects on child’s dietary patterns. Then implementation research could be done to promote the uptake of interventions that have been proven effective into routine practices.

## 5 Conclusions and suggestions

Nearly two-thirds of children in Beijing, China, consume fruit daily, although this frequency is lower among boys and older students. A positive correlation was observed between both individual food and nutrition literacy and family food environment with children’s fruit consumption. It was noteworthy that interventions aimed at increasing fruit intake should focus on boys who were in higher grades, had caregivers with lower educational levels, and had lower family incomes.

Future interventions should prioritize nutrition education in schools, especially in higher grades of middle schools, to promote students’ knowledge acquirement, beliefs generation and food consumption behavior change. Although the present study did not involve the school food environment, school feeding and a healthy school environment establishment are equally important for children’s food consumption behaviors (12). Another effective strategy is to motivate parents to foster a supportive family environment. This can be achieved through positive role modeling and parental feeding practices, ensuring fruit availability, and establishing rules to guide child’s eating behaviors. It should be emphasized that caregiver’s food and nutrition literacy could be acquired by parent-teacher meetings, and positive interaction between family and school is extremely important for children’s behavior and health. Of course, media publicity including food advertisement is also important social environment (8–12).

## Data availability statement

The raw data supporting the conclusions of this article will be made available by the authors, without undue reservation.

## Ethics statement

The studies involving humans were approved by Institutional Review Board of the Beijing Center for Disease Prevention and Control. The studies were conducted in accordance with the local legislation and institutional requirements. Written informed consent for participation in this study was provided by the participants' legal guardians/next of kin.

## Author contributions

XW: Data curation, Writing – original draft. YY: Conceptualization, Data curation, Formal analysis, Investigation, Methodology, Project administration, Resources, Writing – review & editing. HH: Investigation, Writing – original draft. XY: Investigation, Writing – original draft. DG: Investigation, Resources, Writing – original draft. WZ: Conceptualization, Data curation, Methodology, Supervision, Writing – original draft, Writing – review & editing.
